# Atorvastatin promotes bone formation in aged apoE^–/–^ mice through the Sirt1–Runx2 axis

**DOI:** 10.1186/s13018-020-01841-0

**Published:** 2020-08-06

**Authors:** Wei Hong, Zhanying Wei, Zhaohui Qiu, Zheng Li, Chensheng Fu, Zhibin Ye, Xiaoya Xu

**Affiliations:** 1 Department of Osteoporosis and Bone Metabolism Disease, Huadong Hospital, Fu Dan University, Shanghai, China; 2 Shanghai Key Laboratory of Clinical Geriatric Medicine, Huadong Hospital, Fudan University, Shanghai, China; 3 Department of Osteoporosis and Related Bone Disease, Shanghai 6th People’s Hospital, Shanghai Jiao Tong University School of Medicine, Shanghai, China; 4 Department of Cardiology, TongRen Hospital, Shanghai Jiao Tong University School of Medicine, Shanghai, China; 5 Laboratory Animal Center, Zhongshan Hospital, Fudan University, Shanghai, China; 6 Department of Nephrology, Huadong Hospital, Fudan University, Shanghai, China; 7 Department of Radiation Biology, Institute of Radiation Medicine, Fudan University, Shanghai, China

**Keywords:** ApoE^−/−^ mice, Aged, Sirt1, Bone formation, Atorvastatin

## Abstract

**Background:**

Statins are the most widely used drugs in elderly patients; the most common clinical application of statins is in aged hyperlipemia patients. There are few studies on the effects and mechanisms of statins on bone in elderly mice with hyperlipemia. The study is to examine the effects of atorvastatin on bone phenotypes and metabolism in aged apolipoprotein E-deficient (apoE^–/–^) mice, and the possible mechanisms involved in these changes.

**Methods:**

Twenty-four 60-week-old apoE^–/–^ mice were randomly allocated to two groups. Twelve mice were orally gavaged with atorvastatin (10 mg/kg body weight/day) for 12 weeks; the others served as the control group. Bone mass and skeletal microarchitecture were determined using micro-CT. Bone metabolism was assessed by serum analyses, qRT-PCR, and Western blot. Bone marrow-derived mesenchymal stem cells (BMSCs) from apoE^–/–^ mice were differentiated into osteoblasts and treated with atorvastatin and silent information regulator 1 (Sirt1) inhibitor EX-527.

**Results:**

The results showed that long-term administration of atorvastatin increases bone mass and improves bone microarchitecture in trabecular bone but not in cortical bone. Furthermore, the serum bone formation marker osteocalcin (OCN) was ameliorated by atorvastatin, whereas the bone resorption marker tartrate-resistant acid phosphatase 5b (Trap5b) did not appear obviously changes after the treatment of atorvastatin. The mRNA expression of Sirt1, runt-related transcription factor 2 (Runx2), alkaline phosphatase (ALP), and OCN in bone tissue were increased after atorvastatin administration. Western blot showed same trend in Sirt1 and Runx2. The in vitro study showed that when BMSCs from apoE^–/–^ mice were pretreated with EX527, the higher expression of Runx2, ALP, and OCN activated by atorvastatin decreased significantly or showed no difference compared with the control. The protein expression of Runx2 showed same trend.

**Conclusions:**

Accordingly, the current study validates the hypothesis that atorvastatin can increase bone mass and promote osteogenesis in aged apoE^−/−^ mice by regulating the Sirt1–Runx2 axis.

## Background

Osteoporosis (OP) and atherosclerosis (AS) are public health problems that accompany the aging process and have numerous epidemiological links with and economic consequences for the elderly population. Clinical studies have demonstrated that cardiovascular diseases are associated with reduced bone mineral density (BMD) and increased bone fractures [1–4]. These two age-related diseases may be sustained by similar risk factors, for example, oxidative stress, inflammation, free radicals, lipid metabolism, and estrogen deficiency, as well as common pathological mechanisms and molecular mediators, such as the Wnt pathway, receptor activator of nuclear factor kappa B ligand (RANKL), osteoprotegerin (OPG), bone morphogenic protein (BMP), and Sirt1 [5–8].

Additional evidence suggests that a link between AS and OP may be related to their common response to drugs. For example, bisphosphonates, raloxifene, and statins may be effective in treating both AS and OP, which also suggests a common pathophysiological basis [7, 9, 10]. Among these drugs, statins are the most widely used drugs in elderly patients with atherosclerosis. In addition to its anti-atherosclerotic, anti-inflammatory, and antioxidative stress effects, studies have shown that statins can stimulate osteoblast differentiation [11–13] as well as inhibit osteoclast function in vitro [14, 15] through various signaling pathways. However, the effects of statins on osteoporosis and fracture prevention in clinical and animal experiments remain controversial [9, 16–18]. Moreover, most previous studies focused on the effects of statins on ovariectomized (OVX) or fracture models [19–21]. However, the most common clinical application of statins is in aged patients with hyperlipidemia [22, 23]. There are few studies on the effects and mechanisms of statins on bone in elderly mice with hyperlipidemia.

Sirt1, a highly conserved NAD^+^-dependent histone deacetylase, is a vital protein and target for pathways involved in the regulation of lifespan and aging-related diseases [24]. Sirt1 has been shown to be atheroprotective in apoE^–/–^ mice [25, 26]. Recent studies have shown that Sirt1 also plays an important role in the pathogenesis of osteoporosis, as well as in mesenchymal stem cell differentiation [27, 28]. Our previous study found that Sirt1 is involved in decreased bone formation in aged apolipoprotein E-deficient mice. Long-term treatment with oxidized low-density lipoprotein (ox-LDL) decreased the expression of Sirt1 in BMSCs [29]. On the other hand, resveratrol (an agonist of Sirt1) was recently found to mediate the modulation of Sirt1-Runx2, promoting the osteogenic differentiation of mesenchymal stem cells due to its effect on Runx2 deacetylation [30]. Statins have demonstrated antioxidative effects on atherosclerosis through the inhibition of Sirt1 expression [31, 32].

Thus, we first investigated the effects of oral atorvastatin on bone mass and bone formation in aged apoE^–/–^ mice. Then, we further detected how statins regulate osteoblast differentiation in an aged hyperlipidemia mouse model. To test this hypothesis, the effects of atorvastatin on Sirt1 expression in bone and the role of Sirt1-Runx2 in the expression of BMSCs from apoE^−/−^ mice were also evaluated.

## Materials and methods

### Animal experiments

Sixty-week-old male apoE^−/−^ mice with a C57Bl/6 J background were purchased from Nanjing Biomedical Research Institute (Nanjing University, Nanjing, China) and fed with regular rodent chow (containing 20.5% protein, 4.68% fat, 1.23% calcium, and 0.91% phosphorus). Twenty-four mice were randomly allocated to two groups. Twelve mice (atorvastatin group) were orally gavaged with 10 mg kg^**−**1^ day^**−**1^ atorvastatin (Pfizer, New York, USA) dissolved in DMSO (dimethyl sulfoxide) for 12 weeks. The other mice served as the control group and received an equivalent amount of DMSO as placebo. The mice were maintained in groups under a strict 12 h light/dark cycle in a temperature-controlled environment (25 °C) with free access to food and water. The procedures involving animals and their care were conducted in conformity with the guidelines of the department of laboratory animal science of Fudan University. All animal experiments were conducted with the approval of the Animal Resources Committee of Huadong Hospital, Fudan University (2012-03-FYS-GJJ-01).

### Microcomputed tomography (micro-CT) analysis

The femurs collected from apoE^–/–^ mice at 72 weeks of age were harvested, fixed, and scanned by the SkyScan-1176 micro-CT instrument (Bruker, Kontich, Belgium). For cortical bone, measurements were performed on a 0.5-mm region of the mid-diaphysis of the femur. For trabecular bone, micro-CT evaluation was performed on a 1-mm region of the metaphyseal spongiosa on the distal femur. The regions were located 0.5 mm above the growth plate. NR Econ software version 1.6 was used for the three-dimensional (3D) reconstruction and viewing of images [29].

### Biochemical and bone markers assays of serum

Serums from two groups’ mice were analyzed for OCN by enzyme-linked immunosorbent assays (ELISAs) using a Mouse Osteocalcin EIA Kit (Immutopics International, San Clemente, CA, USA) and for TRAP5b using MouseTRAP^TM^ [TRAcP5b] ELISA (Immunodiagnostic Systems Limited, London, England) serum total cholesterol (TC) was measured by a Hitachi 917 autoanalyzer (Roche, Meylan, France), and ox-LDL was measured by a mouse ox-LDL ELISA kit (Uscn Life Science & Technology, Wuhan, China) [29].

### Cell culture

BMSCs were obtained from the femurs and tibias of apoE^–/–^ mice (male, 18 weeks old). The second generation of cells was seeded at 1 × 10^5^ cells/well in a six-well culture plate and cultured in L-DMEM containing 10% fetal bovine serum.

(1) BMSCs were cultured with atorvastatin at different concentrations (0, 10, 100, 1000 nmol/L) at 6 h for qRT-PCR and at 3 days for Western blot. (2) BMSCs were cultured with EX-527 (Sigma, USA) at a concentration of 10 μM for 2 h; then, the medium was changed to atorvastatin (100 nmol/L) at 6 h for qRT-PCR and at 3 days for Western blot.

### Quantitative real-time reverse transcription-polymerase chain reaction (qRT-PCR)

Total RNA was extracted from tissues and cultured BMSCs. Real time PCR was conducted as previously described [29]. The primers were obtained from SBSgene (www.sbsgene.com) with the following primer sequences: Sirt1, 5′-GCA GGT TGC AGG AAT CCA A-3′ and 5′-GGC AAG ATG CTG TTG CAA A-3′ (63 bp, XM_006514342.1); runt-related transcription factor 2 (Runx2), 5′-TGT TCT CTG ATC GCC TCA GTG-3′ and 5′-CCT GGG ATC TGT AAT CTG ACT CT-3′ (146 bp, XM_006523545.1); ALP, 5′-CAC GCG ATG CAA CAC CAC TCA GG-3′ and 5′-GCA TGT CCC CGG GCT CAA AGA-3′ (479 bp, XM_ 006538500.1); osteocalcin (OCN), 5′-ACC CTG GCT GCG CTC TGT CTC T-3′ and 5′-GAT GCG TTT GTA GGC GGT CTT CA-3′ (240 bp, NM_007541.3); and glyceroldehyde -3-phosphate dehydrogenase (GAPDH), 5′-AGC CTC GTC CCG TAG ACA-3′ and 5′-CTC GCT CCT GGA AGA TGG-3′ (255 bp, NM_008084.3).

### Western blot analysis

All samples were collected, and the Western blot was conducted as previously described [29]. The primary antibodies used were rabbit anti-Sirt1 (Cat. No: 13161-1-AP; 1:2000; Proteintech, Rosemont, USA), mouse anti-Runx2 (Cat. No: ab54868; 1:1000; Abcam, CA, USA), and mouse anti-GAPDH (Cat. No: KC-5G4; 1:10,000; Kangcheng, Shanghai, China). The protein bands were quantitatively analyzed using an image analysis system (Quantity One software; Bio-Rad, Hercules, CA, USA).

### Statistical analysis

All data are presented as the mean ± standard deviation. We used two-tailed *t* tests to determine the significance between two groups. We analyzed the results of multiple groups by two-way ANOVA with Bonferroni post hoc tests using the SPSS version 17 software. For all statistical tests, we considered a *p* value < 0.05 statistically significant.

## Results

### Atorvastatin increases trabecular bone volume and bone formation in aged apoE^−/−^ mice

**Fig. 1:**
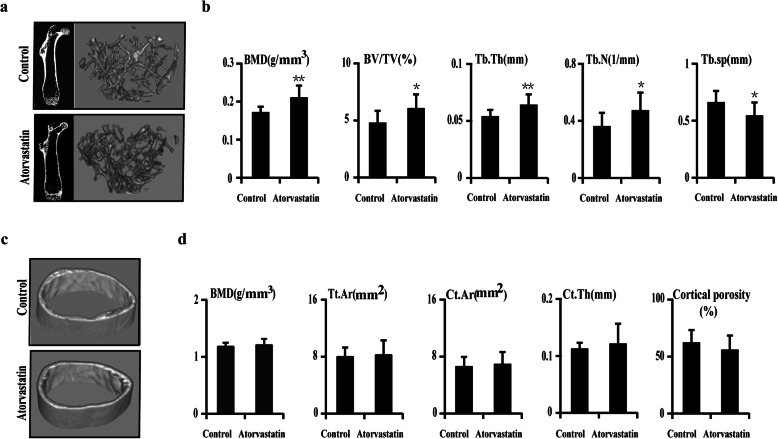
The effect of atorvastatin (10 mg/kg/day) treatment on bone volume in aged apoE^−/−^ mice, as determined by micro-CT. **a** Representative 3D micro-CT images showing regions of the trabecular bone in the distal femurs. **b** The graphs represent measurements of bone mineral density (BMD), trabecular bone volume (BV/TV), trabecular thickness (Tb.Th), trabecular number (Tb.N), and trabecular spacing (Tb.Sp) in the femurs from control and atorvastatin groups. **c** Representative 3D micro-CT images showing regions of cortical bone in the femurs from control and atorvastatin groups. **d** The graphs represent measurements of BMD, total area (Tt.Ar), cortical area (Ct.Ar), cortical thickness (Ct.Th), and cortical porosity. *N* = 12 mice per group were examined. *p < 0.05, **p < 0.01, vs. control group

To determine the effects of atorvastatin on the bone phenotype in aged apoE^−/−^ mice, we first performed micro-CT reconstruction on the distal metaphyseal region of the femurs (Fig. 1). Relative to the control group, femurs from the atorvastatin group exhibited marked increases in trabecular BMD (+22.57%, *p* < 0.01), trabecular bone volume (BV/TV, +26.93%, *p* < 0.05), trabecular thickness (Tb.Th, +19.25%, *p* < 0.01), trabecular number (Tb.N, +31.24%, *p* < 0.05), and decreased trabecular spacing (Tb.sp, −17.87%, *p* < 0.05) (Fig. 1a, b) after adjustment for body weight. No significant changes in the cortical parameters of femurs were observed between the atorvastatin and control groups (Fig. 1c, d).

### Atorvastatin improved the balance of bone turnover in aged apoE^−/−^ mice

**Fig. 2:**
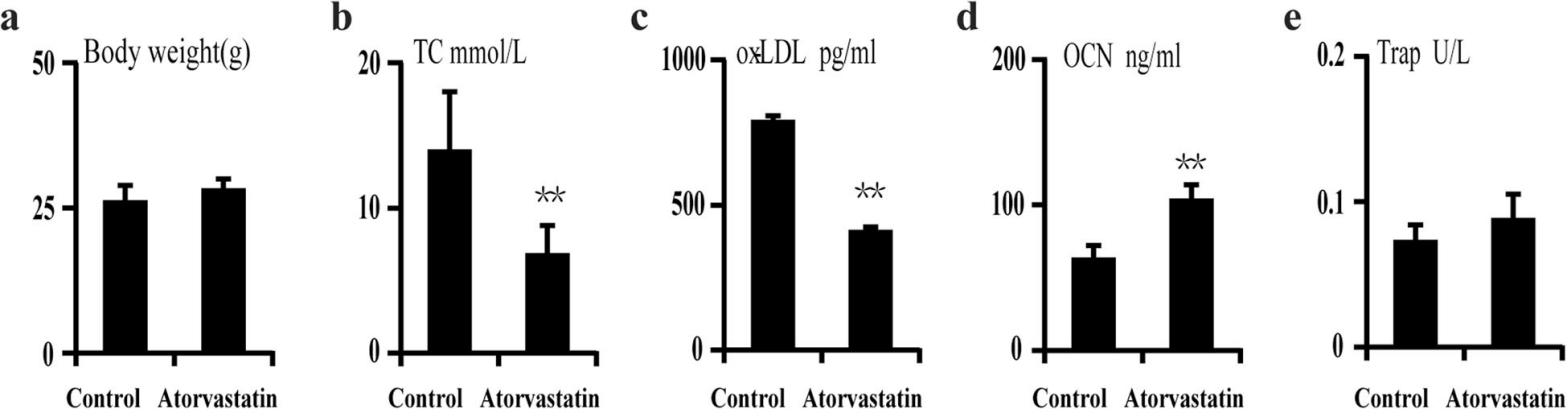
The effect of atorvastatin (10 mg/kg/d) treatment on bone turnover markers changes in aged apoE^−/−^ mice. **a** The body weight of two groups. **b** The level of serum total cholesterol (TC) in two groups. **c** The level of oxidized low-density lipoprotein (ox-LDL) in two groups. **d** The change of serum of osteocalcin (OCN) in two groups. **e** The change of serum of tartrate-resistant acid phosphatase 5b in two groups. *N* = 6 mice per group were examined. *p < 0.05, **p < 0.01, vs. control group

To further understand the changes in bone turnover in aged apoE^−/−^ mice after treatment with atorvastatin, the levels of serum TRAP5b and OCN were also measured between the two groups. Compared to the control group, the level of serum OCN significantly increased (+66.2%, **p < 0.01, Fig. 2d) in aged apoE^−/−^ mice treated with atorvastatin, accompanied by a decrease in serum total cholesterol (TC, −51.6%, **p < 0.01, Fig. 2b) and oxidized low-density lipoprotein (ox-LDL, −48.4%, **p < 0.01, Fig. 2c). There were no remarkable changes in body weight or serum tartrate-resistant acid phosphatase 5b (TRAP5b) between the two groups (Fig. 2a, e).

### Atorvastatin increased Sirt1 expression in the bone tissue of aged apoE^−/−^ mice

**Fig. 3:**
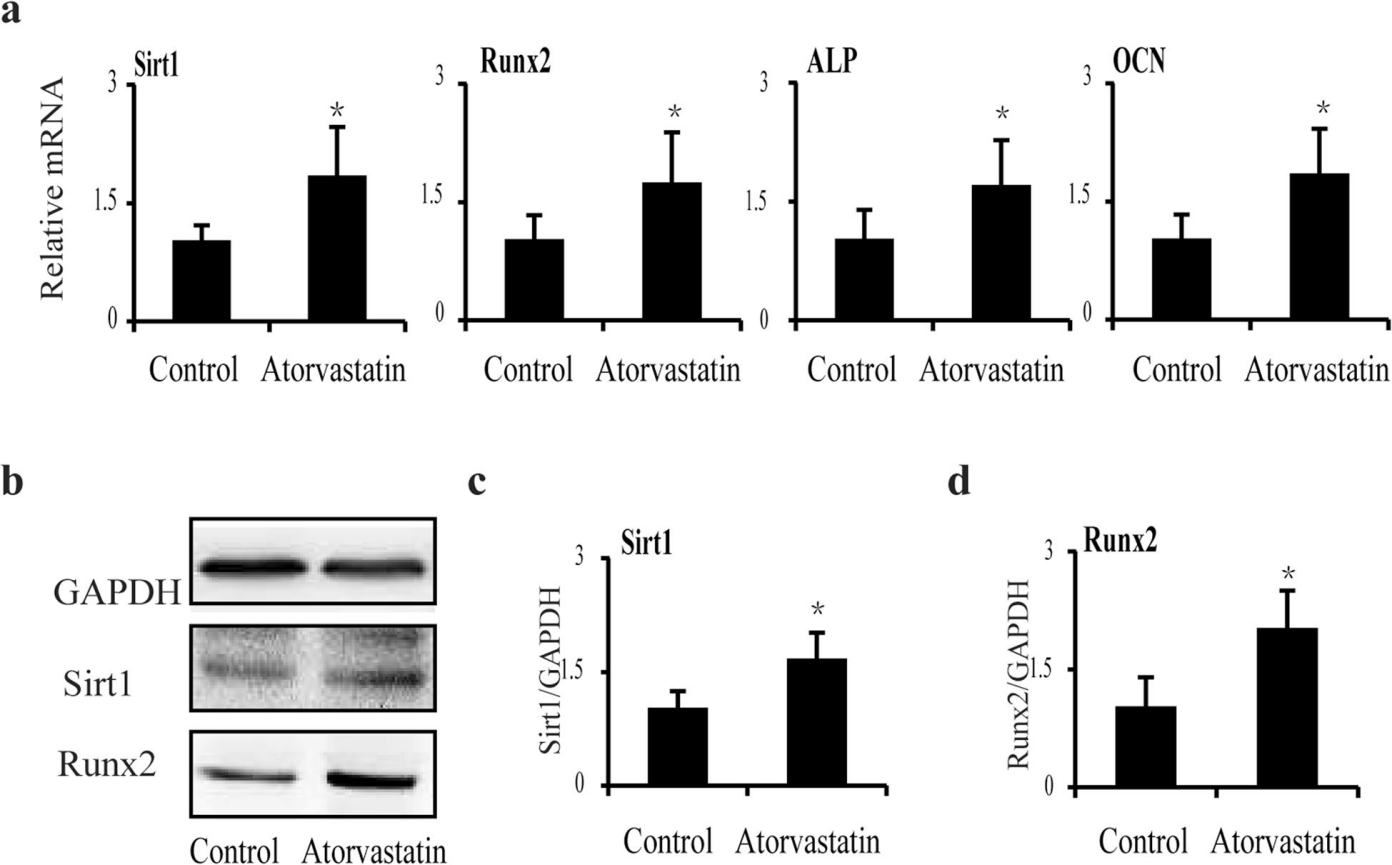
The gene and protein expressions of bone formation in bone tissue from aged apoE^–/–^ mice after atorvastatin treatment. **a** mRNA expression of Sirt1, Runx2, ALP, and OCN in whole femurs from two groups. **b**–**d** Western blot showed levels of Sirt1 and Runx2 from whole femurs in two groups. *N* = 3 mice per group were examined. *p < 0.05 vs. control group

To further analyze the mechanisms of the increase in bone mass and bone formation in aged apoE^−/−^ mice after atorvastatin treatment, we measured Sirt1 and Runx2 expression in the bone tissue of the two groups (Fig. 3). When aged apoE^−/−^ mice were treated with atorvastatin (10 mg kg^**−**1^ day^**−**1^) for 12 weeks, the mRNA expression of Sirt1 in bone tissue increased by 82.1% compared with expression in the control group (*p* < 0.05) (Fig. 3a). Similarly, the mRNA expression of Runx2, ALP, and OCN was significantly higher in the atorvastatin group after normalization (+72.6%, +68.6%, and + 82.8%, respectively, *p* < 0.05) (Fig. 3a). Western blot analysis showed that the levels of Sirt1 and Runx2 increased by 65.8% and 105.6% in the atorvastatin group compared to the control group (*p* < 0.05) (Fig. 3b-d).

### Atorvastatin increases the expression of Sirt1 and Runx2 in BMSCs from apoE^−/−^ mice in vitro

**Fig. 4:**
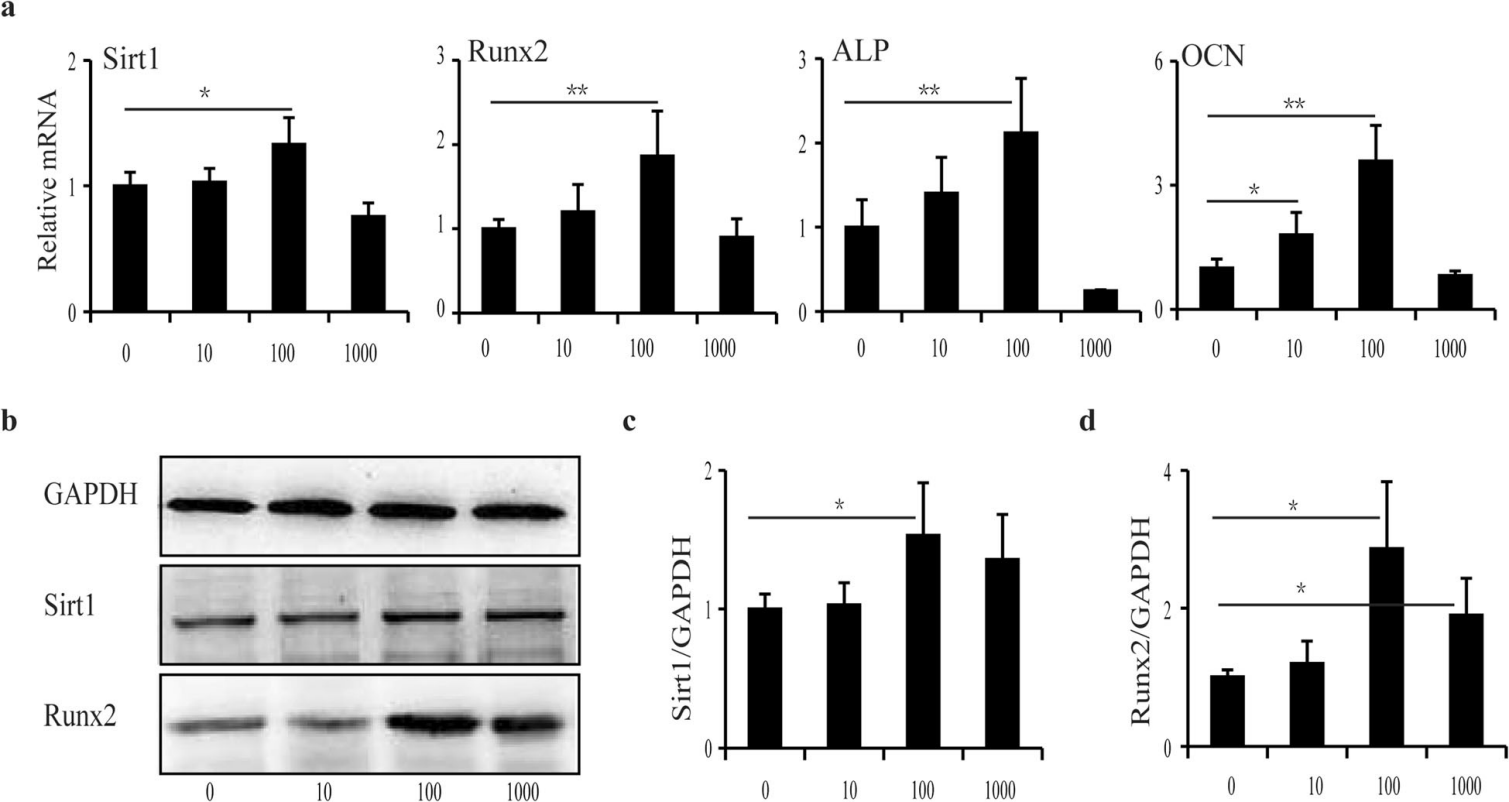
The expression of Sirt1, Runx2, ALP, and OCN in BMSCs from apoE^–/–^ mice treated with 10, 100, and 1000 nmol/L atorvastatin in vitro. **a** The mRNA expression of Sirt1, Runx2, ALP, and OCN in BMSCs with different concentrations of atorvastatin. **b**–**d** The protein expression of Sirt1 and Runx2 in BMSCs with different concentrations of atorvastatin. *N* = 3. *p < 0.05, **p < 0.01 vs. 0 nmol/L group

To comprehensively determine the mechanism for atorvastatin-induced osteogenesis in apoE^−/−^ mice, we investigated the effect of atorvastatin on the expression of Sirt1 and Runx2 in BMSCs in vitro. We treated BMSCs from 18-week-old apoE^−/−^ mice exposed to different concentrations of atorvastatin (0, 10, 100, and 1000 nmol/L). The results showed that the mRNA and protein expression of Sirt1 and Runx2 increased significantly with the concentration of atorvastatin, reaching a maximum at a concentration of 100 nmol/L (*p* < 0.05 and *p* < 0.01, compared to 0 nmol/L) (Fig. 4a). The mRNA expression of ALP and OCN in BMSCs showed the same trend (*p* < 0.01) (Fig. 4a). The protein expression of Sirt1 and Runx2 also increased significantly with the concentration of atorvastatin, reaching a maximum at a concentration of 100 nmol/L (*p* < 0.05, compared to 0 nmol/L) (Fig. 4b-d).

### Atorvastatin promoted osteogenesis in BMSCs from apoE^−/−^ mice through activation of Sirt1

**Fig. 5:**
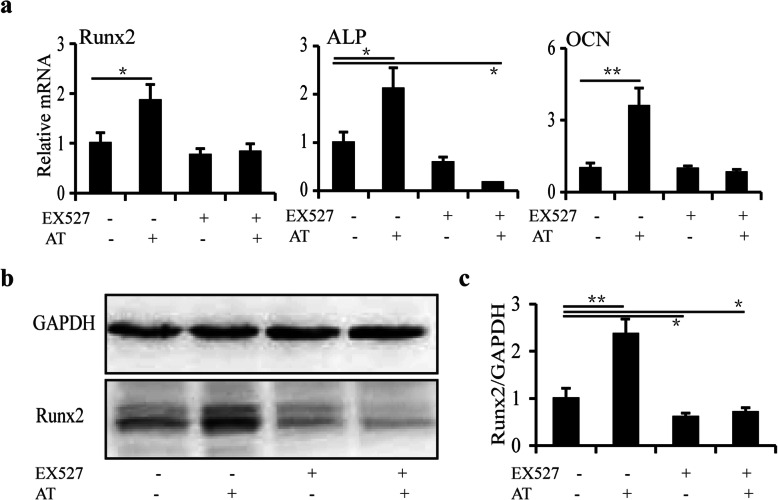
Sirt1-Runx2 is mainly involved in atorvastatin-induced osteogenesis in BMSCs from apoE^–/–^ mice. **a** The mRNA expression of Runx2, ALP, and OCN in the presence of 100 nmol/L atorvastatin with or without the treatment of EX527. **b**, **c** The protein expression of Runx2 in BMSCs from apoE^–/–^ mice in the presence of 100 nmol/L atorvastatin with or without the treatment of EX527. *N* = 3. *p < 0.05, **p < 0.01 vs. control group. AT, atorvastatin

Based on the preceding observations, we then searched for the mechanism underlying the effect of atorvastatin on Sirt1–Runx2-mediated osteogenesis. The mRNA expression of Runx2, ALP, and OCN in BMSCs increased significantly (+86.1%, +111.7%, and + 258.9%, respectively, *p <* 0.05, *p <* 0.01) after atorvastatin (100 nmol/L) treatment. However, when the BMSCs were pre-treated with EX527 (10 μM), the atorvastatin-induced increase in the mRNA expression of Runx2, ALP, and OCN was abolished and even decreased significantly (*p <* 0.05) or showed no difference (Fig. 5a). The protein expression of Runx2 increased by 136.1% after atorvastatin treatment. When BMSCs were pre-treated with EX527, the expression of Runx2 decreased by 15.2% and 18.9% after treatment without or with atorvastatin, respectively, compared with the control (*p* < 0.05) (Fig. 5b, c).

## Discussion

Statins, the most commonly used lipid-lowering drugs, are routinely administered to treat hyperlipidaemia in the elderly population. In the last two decades, evidence has shown that statins may be beneficial as a potential anti-osteoporosis drug for bone regeneration [11–13]. However, most previous studies focused on statins’ effects on OVX animal or fracture animal models [19, 33–36]. The majority of related studies showed an increase in BMD or the promotion of osteogenic differentiation after statin treatment [35–39], while other studies detected a negative association between statin treatment and BMD and bone repair [40–42]. The role of statins in these animal model studies remains controversial.

To the best of our knowledge, there have been few studies on the effects of statins on bone in aged hyperlipidemia mouse models. apoE^–/–^ mice are characterized by severe hyperlipidemia and the spontaneous development of atherosclerosis [43]. In our previous results, aged apoE^–/–^ mice displayed low bone mass and decreased bone formation due to an ox-LDL effect on Sirt1 and Runx2 in BMSCs. Thus, in this study, we investigated the bone phenotypes and metabolism of aged apoE^–/–^ mice treated with oral atorvastatin and attempted to elucidate the possible mechanisms involved in these changes. Compared with the control group, after 3 months of oral treatment with therapeutic doses of atorvastatin, aged apoE^–/–^ mice demonstrated an increase in trabecular bone volume but not cortical bone volume. Furthermore, serum bone OCN increased significantly in aged apoE^–/–^ mice after atorvastatin treatment. Serum osteocalcin levels were correlated with bone formation and osteoblast number. As a product of osteoblastic synthesis, osteocalcin has been used as a marker of bone formation. On the other hand, the levels of the bone resorption marker Trap5b did not change significantly. These results indicate that atorvastatin could act as a bone anabolic agent to increase bone mass in aged apoE^−/−^ mice. Recently, a randomized controlled study focused on the effects of statins on BMD in elderly males with osteopenia and mild dyslipidaemia [22]. Consistent with our findings, oral treatment with therapeutic doses of atorvastatin was associated with significantly higher values of total hip BMD in elderly men compared with the control group after a 1-year study period.

Several mechanisms of action of statins on bone have been demonstrated in previous studies. For example, statins induce osteoblastic differentiation by upregulating the MAPK/BMP-2 signaling pathway [44]. Statins inhibit osteoblastic apoptosis by the TGFβ/Smad3 signaling pathway [45]. In an aging population, more factors may be involved. Sirt1 plays important roles in the progression of the age-related diseases atherosclerosis and osteoporosis [46–48]. Our previous study found low Sirt1 expression in the bone tissue of aged apoE^−/−^ mice compared with age-matched wild-type mice [29]. Oxidized LDL is also an important contributor to the aging process. Ox-LDL levels gradually increase with age. The long-term accumulation of ox-LDL-induced Sirt1 decreases in bone marrow-derived mesenchymal stem cells. This may have contributed to the bone loss in the aged apoE^−/−^ mice seen in our previous study [29]. Statins have been proven to have a preventive effect on aging through intervention with Sirt1 [49]. Statins could inhibit endothelial senescence and apoptosis through the upregulation of Sirt1 [50]. Long-term administration of atorvastatin to old rats inhibited the senescent phenotype through increased Sirt1 expression, improved endothelium-dependent relaxation, and ameliorated oxidative stress [51]. However, to the best of our knowledge, no study has reported the effects of statins on Sirt1 in bone. In the present study, after 3 months of intervention with oral atorvastatin, the expression levels of Sirt1 and Runx2 were significantly higher than those of the control group. Runx2 is the most upstream transcription factor essential for osteoblast differentiation [52]. Studies have shown that Sirt1 mediates Runx2 deacetylation, promoting the osteogenic differentiation of mesenchymal stem cells. Resveratrol, which is an agonist of Sirt1, promotes the osteogenesis of human mesenchymal stem cells by upregulating Runx2 gene expression via the Sirt1/Foxo3A axis. The activation of Sirt1 using resveratrol and isonicotinamide was shown to stimulate osteoblastic differentiation in a dose-dependent manner by increasing the mRNA expression of Runx2, ALP, and OCN [53]. Based on these findings, we hypothesize that atorvastatin could promote osteogenesis in the BMSCs of apoE^−/−^ mice via enhancement of the Sirt1–Runx2 pathway [54]. To further confirm this hypothesis in more detail, we measured the expression levels of Sirt1 and Runx2 in BMSCs from apoE^−/−^ mice after stimulation with atorvastatin in vitro. qRT-PCR and Western blot analyses showed that atorvastatin enhanced Sirt1 and Runx2 expression in BMSCs from apoE^−/−^ mice, whereas atorvastatin-induced Sirt1 and Runx2 activity was totally abolished by pre-treatment with the Sirt1 inhibitor EX527 (10 μM). The results showed that atorvastatin may promote bone formation in BMSCs from apoE^−/−^ mice via the Sirt1–Runx2 axis.

## Conclusions

In this study, we investigated the bone phenotypes and metabolism of aged apoE^–/–^ mice treated orally with atorvastatin and attempted to elucidate the possible mechanisms involved in these changes. Our results showed that long-term administration of atorvastatin increased trabecular bone mass and promoted bone formation in aged apoE^−/−^ mice. Our data also showed that the mechanism of atorvastatin might promote the osteogenesis of BMSCs from apoE^−/−^ mice by upregulating the Sirt1–Runx2 axis.

## Data Availability

We declare that the materials described in the manuscript will be freely available to all scientists for noncommercial purpose.

## Abbreviations

ALP: Alkaline phosphatase
AT: Atorvastatin
AS: Atherosclerosis
BMSCs: Bone marrow-derived mesenchymal stem cells
BMD: Bone mineral density
BMP: Bone morphogenic protein
BV/TV: Bone volume/total volume
Ct.Ar: Cortical area
Ct.Th: Cortiacal thickness
DMSO: Dimethyl sulfoxide
OCN: Osteocalcin
OPG: Osteoprotegerin
OVX: Ovariectomized
ox-LDL: Oxidized low-density lipoprotein
RANKL: Receptor activator of nuclear factor kappa B ligand
Runx2: Runt-related transcription factor 2
Sirt1: Silent information regulator 1
Tb.Ar: Total area
Tb.N: Trabecular number
Tb.Sp: Trabecular spacing
Tb.Th: Trabecular thickness
TC: Total cholesterol
Trap5b: Tartrate-resistant acid phosphatase 5b
